# Exploring Clinical Associations Using ‘-Omics’ Based Enrichment Analyses

**DOI:** 10.1371/journal.pone.0005203

**Published:** 2009-04-13

**Authors:** David A. Hanauer, Daniel R. Rhodes, Arul M. Chinnaiyan

**Affiliations:** 1 Department of Pediatrics, University of Michigan, Ann Arbor, Michigan, United States of America; 2 Comprehensive Cancer Center, University of Michigan, Ann Arbor, Michigan, United States of America; 3 Center for Computational Medicine and Biology, University of Michigan, Ann Arbor, Michigan, United States of America; 4 Department of Pathology, University of Michigan, Ann Arbor, Michigan, United States of America; 5 Michigan Center for Translational Pathology, University of Michigan, Ann Arbor, Michigan, United States of America; 6 Howard Hughes Medical Institute, Chevy Chase, Maryland, United States of America; University of the Western Cape, South Africa

## Abstract

**Background:**

The vast amounts of clinical data collected in electronic health records (EHR) is analogous to the data explosion from the “-omics” revolution. In the EHR clinicians often maintain patient-specific problem summary lists which are used to provide a concise overview of significant medical diagnoses. We hypothesized that by tapping into the collective wisdom generated by hundreds of physicians entering problems into the EHR we could detect significant associations among diagnoses that are not described in the literature.

**Methodology/Principal Findings:**

We employed an analytic approach original developed for detecting associations between sets of gene expression data, called Molecular Concept Map (MCM), to find significant associations among the 1.5 million clinical problem summary list entries in 327,000 patients from our institution's EHR. An odds ratio (OR) and p-value was calculated for each association. A subset of the 750,000 associations found were explored using the MCM tool. Expected associations were confirmed and recently reported but poorly known associations were uncovered. Novel associations which may warrant further exploration were also found. Examples of expected associations included non-insulin dependent diabetes mellitus and various diagnoses such as retinopathy, hypertension, and coronary artery disease. A recently reported association included irritable bowel and vulvodynia (OR 2.9, p = 5.6×10^−4^). Associations that are currently unknown or very poorly known included those between granuloma annulare and osteoarthritis (OR 4.3, p = 1.1×10^−4^) and pyloric stenosis and ventricular septal defect (OR 12.1, p = 2.0×10^−3^).

**Conclusions/Significance:**

Computer programs developed for analyses of “-omic” data can be successfully applied to the area of clinical medicine. The results of the analysis may be useful for hypothesis generation as well as supporting clinical care by reminding clinicians of likely problems associated with a patient's existing problems.

## Introduction

The implementation of electronic health records (EHR) at our institution and peer institutions has allowed for the storage of vast amounts of information in clinical data repositories. The EHR allows clinicians to maintain a problem summary list (PSL) for each patient which is used in clinical medicine to provide a concise overview of the significant medical issues and diagnoses. Clinicians are free to add whatever problems are deemed appropriate, including both chronic and acute conditions. Like much of the data in the EHR, the items in our PSL are free text, resulting in marked variability. While the PSL is meant primarily for diagnoses, clinicians also often add signs (e.g., fever, tachypnea, pallor) and symptoms (e.g., fatigue, back pain, cough). This has made large-scale analyses and mining of the data a challenge.

Nevertheless, with over 10 years of clinical data in our EHR, we hypothesized that harnessing the power of the roughly 2,000 clinicians in our health system who enter diagnoses in the PSL could help bring to light interesting associations that are either poorly known or unknown. Studies seeking associations among diagnoses in EHRs have been explored in the past, although they have often focused on specific diseases[1], [2] or used coded concepts. [3] One prior study extracted diseases and findings from patient documents using natural language processing to look for associations.[4]

The advent of the “-omics” revolution has led to the development of many software packages for analyzing gene expression data, including a locally developed tool, the Molecular Concept Map (MCM).[5] The MCM application was originally developed to perform analyses of gene expression data to find significant associations among gene expression signatures. MCM also has the ability to construct network graphs of associations which allows for visualization of the relationship to help answer why two concepts may be associated. Another analogous approach is gene set enrichment analysis developed by the Broad Institute.[6], [7] Fortunately, MCM is flexible enough to accommodate other data types including free text clinical data, making it an ideal platform for exploratory studies using data from the EHR.

## Methods

To test our hypothesis we chose to use an unbiased approach to look for co-occurrences among all entries in the PSL. We combined the automated processes supported by the MCM application with manual human interpretation of the results.

After receiving approval from our institutional review board we obtained 1.5 million free text problem summary list diagnoses for approximately 327,000 patients in our clinical data repository. A total of 20,705 unique free text diagnoses that each appeared in at least 5 patients were included. Some of the most common diagnoses included “hypertension”, “infection”, “depression”, “asthma”, “otitis media”, and “diabetes”, with 58,110, 31,044, 29,025, 28,864, 27,863, and 27,410 instances of each, respectively.

The MCM application was capable of automatically mapping smaller terms that were subsets of larger ones (e.g., “type 2 diabetes” into “type 2 diabetes mellitus”). Due to the variability in the wording of the free text diagnoses, we manually reviewed the 3,500 most common terms in our list of 20,705. For terms that were abbreviated, we manually mapped them to one another so that, for example “T2DM” was made equivalent to “type 2 diabetes mellitus”. We stopped manual mapping after 3,500 terms because most terms at that point were considered unique or already mapped to another term. Of these 3,500 most common terms we mapped 330 common diagnoses, some of which were actually variations of the same concept (e.g., “GIB” = “GI bleed” = “gastrointestinal bleed”).

These data were then loaded into the MCM application for further analysis. Each patient and his or her associated diagnoses was considered to be equivalent to a gene expression signature. Pairwise associations were computed across all clinical problems from the PSL. Odds ratios (ORs) and p-values were calculated for each association.

We then used the graphical user interface of MCM to search for both common and unusual associations. Common associations were sought to provide internal validity to the findings of the system, since we expected that well-known associations would be uncovered. This process was performed manually by typing a diagnosis into MCM and then reviewing the significantly associated diagnoses discovered by the system. We also looked for unknown, or poorly known, associations and then sought confirmation for these associations in the literature with a PubMed search. No comprehensive database of all known clinical associations is available for comparison, which is why our process of validation and data exploration was manual.

## Results and Discussion

**Figure 1:**
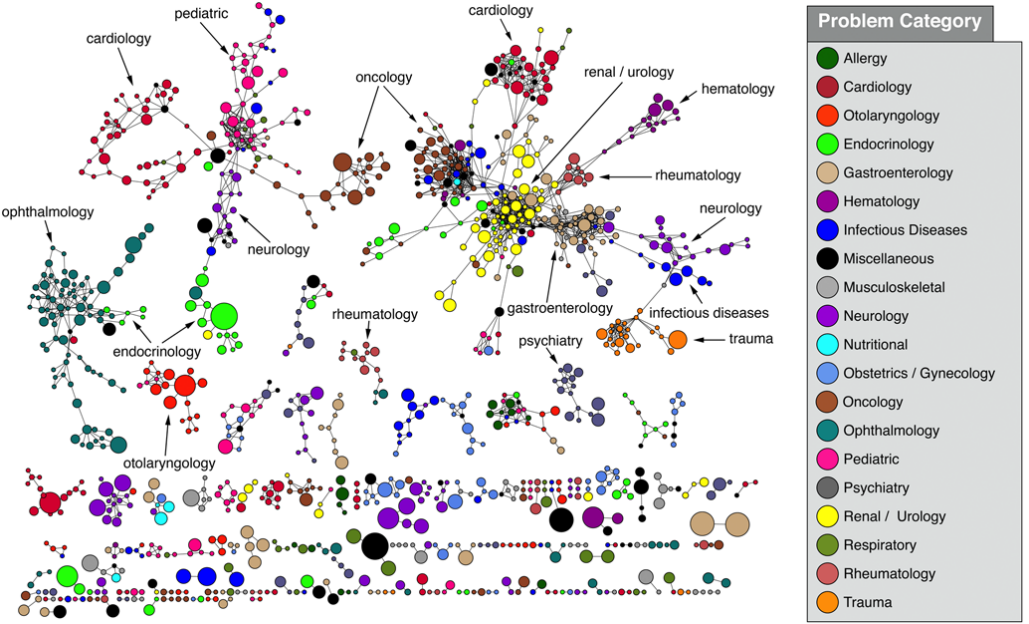
Overall network diagram containing 1106 nodes and 1939 edges showing the most significant problem category associations using an odds ratio>100.0 and p-value<1.0×10^−10^ as thresholds for inclusion. Nodes are roughly proportional to the number of times each problem appears in the problem summary list (PSL) and only nodes with more than 100 occurrences are shown. Problems are color-coded based on the general area in medicine in which the problem would likely be diagnosed or followed. At this level several clusters of related problems can be seen, some of which are labeled above.

We explored numerous associations among diagnoses in our electronic medical record using the Molecular Concept Maps (MCM) web application. The analysis uncovered 753,574 associations among the problems, of which 483,802 associations had an odds ratio greater than 3.0 and a p-value less than 1.0×10^−3^. These associations represented just 0.2% of the possible pairs based on the original list of 20,705 problems. A network graph with the strongest associations is shown in Figure 1. Clusters of diagnoses within similar medical categories can be seen in this high-level view.

**Figure 2:**
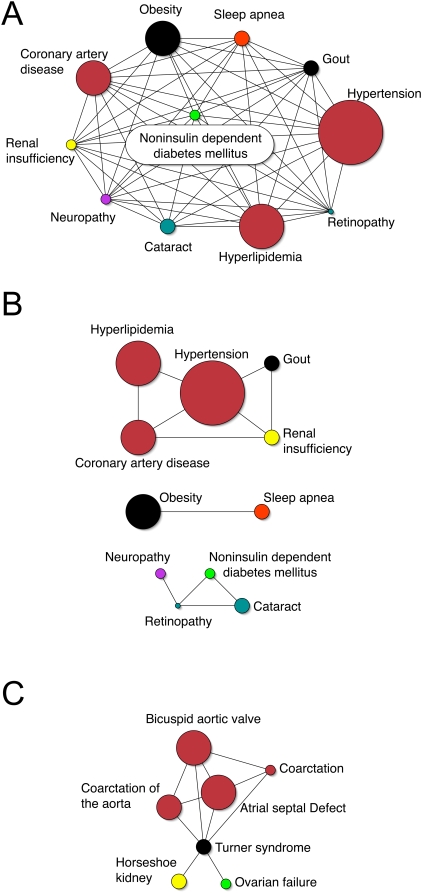
Network graphs showing well-known clinical associations. Node size represents the approximate number of diagnoses in the database and edges represent significant associations between nodes. Node colors are designated according to the legend in Figure 1. 2A displays the complex network of associations linked to the diagnosis of “noninsulin dependent diabetes mellitus” (type 2 diabetes mellitus) using an odds ratio of 1.25 or greater. While the network is mostly interconnected, “cataracts” are not directly associated with either “obesity” or “sleep apnea”. 2B displays the same diagnoses associated with NIDDM using an odds ratio of 8.0 or more as a threshold for connections between nodes. At this odds ratio less significant associations drop out and stronger ones persist. 2C shows common associations with the diagnosis “Turner syndrome” using an odds ratio of 1.25 or greater. “Horsehoe kidney” and “ovarian failure” are independently associated with Turner syndrome, whereas the cardiac defects are associated with one another. Coarctation appears twice because of the free text variability of the diagnoses.

Many of the associations we found were already well known; selecting those which were noteworthy for exploration required a background in clinical medicine. The associations in Figure 2 are generally well known and provided us with validation that the tool adequately discovered significant and expected associations. This is true for both the common diagnosis of non-insulin dependent diabetes mellitus (type 2 diabetes) as well as the less common diagnosis of Turner syndrome. Diagnoses associated with Turner syndrome included frequently described defects such as coarctation of the aorta (OR 140.0, p = 6.4×10^−10^), horseshoe kidney (OR 322.5, p = 1.1×10^−11^), and ovarian failure (OR 155.1, p = 1.4×10^−6^).[8] Several more well known associations are shown in Table 1.

**Table 1:** Well-known associations among problems in the PSL, including supporting literature.

Problem	Associated problem	P value	Odds ratio	Supporting Literature (PubMedID)
Low back pain	Insomnia	7.9×10^−77^	4.5	15033151
Henoch Schonlein purpura	Intussusception	1.8×10^−16^	213.4	18351468
Primary sclerosing cholangitis	Ulcerative colitis	1.0×10^−100^	229	18200656
Developmental delay	Phenylketonuria	8.5×10^−5^	41.0	16763886
Secondary hyperparathyroidism	Anemia	1.0×10^−100^	49.6	18496265

Problems in the first column were selected in the MCM application and noteworthy associated problems were explored, reported in the second column.

**Figure 3:**
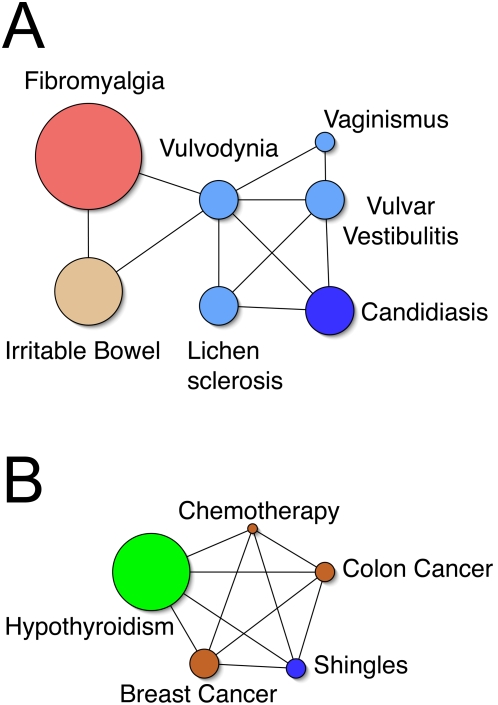
Examples of network graphs used to help identify unexpected associations and form hypotheses about the meaning of the associations. Figure 3A shows a network graph with selected associations for the diagnosis “vulvodynia” using a threshold for edges as odds ratio of 2.5 or more and p-value of 1.0×10^−3^ or less. “Fibromyalgia” and “irritable bowel” are associated with “vulvodynia” independently from the other inter-related gynecologic diagnoses. Figure 3B displays a network graph showing the associations between “shingles”, “hypothyroidism”, and other cancer-related diagnoses, using a threshold for edges as odds ratio of 1.75 or more and p value of 1.0×10^−4^ or less. Use of such a network helps to determine that the relationship between “shingles” and “hypothyroidism” may be due to cancer therapies. Node size represents the approximate number of diagnoses in the database. Node colors are designated according to the legend in Figure 1.

We used the MCM network graphs to identify unexpected associations and form hypotheses about why such associations might exist. Significant associations with the diagnosis of “vulvodynia” are shown in Figure 3A. While most of the associations in the network are related to gynecology, which would be expected, both “irritable bowel” (OR 2.9, p = 5.6×10^−4^), and “fibromyalgia” (OR 5.0, p = 2.5×10^−5^) are not. Two recent articles by Arnold et al reported associations between vulvodynia and both irritable bowel (ORs 1.86 and 3.11) and fibromyalgia (ORs 2.15 and 3.84 ).[9], [10] This compares reasonably well with our findings in MCM.

More associations with recent literature support are in Table 2 and show that MCM revealed associations that have recently been reported. Some of these may be indirect associations. For example, “von Willebrands disease” and “seizure” (OR 5.8, p = 3.4×10^−4^) are likely related because a common medication to treat seizures, valproic acid, has been shown to be a cause of von willebrands disease.[11] Likewise, it is possible that “guillain barre syndrome” is associated with “end stage renal disease” (OR 20.3, p = 6.5×10^−5^) because a common treatment of severe Guillain-Barré syndrome is intravenous immunoglobulins which itself can cause renal failure.[12]

**Table 2:** Recently reported associations with support from the literature.

Problem	Associated problem	P value	Odds ratio	Supporting Literature (PubMedID)
Amyotrophic lateral sclerosis	History of smoking	8.6×10^−22^	101.4	15229114, 10364720
Menieres disease	Hypothyroidism	7.4×10^−12^	4.1	14967756
Intussusception	Herpangina	2.4×10^−4^	26.6	10493041
Pituitary microadenoma	Irritable bowel	4.7×10^−4^	5.5	16472586
Hypothyroidism	Fibromyalgia	7.5×10^−80^	3.8	17102943, 15468372
Migraine headaches	Depression	1.4×10^−100^	3.6	16483117
Acute appendicitis	Nicotine addiction	4.4×10^−4^	8.2	9950450
Migraine headaches	Asthma	2.5×10^−72^	2.2	12236275
Peyronie's disease	Alcoholism	8.0×10^−3^	7.6	16469028
Conduct disorder	Strep	1.5×10^−6^	7.9	11929370, 12880661
Vulvodynia	Fibromyalgia	2.5×10^−5^	5.0	17306651
Vulvodynia	Irritable bowel	5.6×10^−4^	2.9	17306651
Vulvodynia	Candidiasis	8.0×10^−13^	19.2	17306651
Carpal tunnel syndrome	Osteoarthritis	1.2×10^−100^	5.9	12928223
Carpal tunnel syndrome	Diabetes	1.3×10^−48^	2.7	12928223
Carpal tunnel syndrome	Hypothyroidism	4.7×10^−48^	3.1	12928223
Carpal tunnel syndrome	Rheumatoid arthritis	6.2×10^−27^	4.5	12928223
Gout	Cardiomyopathy	1.0×10^−100^	10.5	2256745, 10232447
Tourette syndrome	Migraines	5.0×10^−3^	4.7	14623732
Lyme disease	Depression	4.8×10^−7^	3.79	7943444, 10918770
Diabetes	Tobacco	2.6×10^−56^	1.9	16603565
Schizophrenia	Diabetes	3.0×10^−13^	2.0	15056604
Von willebrands disease	Seizure	3.4×10^−4^	5.8	11913569
Guillain Barre syndrome	End stage renal disease	6.5×10^−5^	20.3	9761533, 9170022

Use of the network graph to reveal plausible explanations for unexpected associations is demonstrated in Figure 3B. When an association between “hypothyroidism” and “shingles” (OR 2.9, p = 6.2×10^−12^) was first noted, a reasonable explanation could not be found. However, adding other significantly associated elements into the network graph provided the likely scenario that both were related to one another as a side effect of chemotherapy or other anti-neoplastic therapies for both breast and colon cancer.

Other unusual associations for which an explanation likely exists are shown in Table 3. The association between “gilberts disease” and “family history of colon cancer” (OR 26.5, p = 2.5×10^−4^) likely exists due to a cancer trial protocol at our institution asking clinicians to monitor bilirubin levels but has exceptions for patients with Gilberts. Thus, the association may simply be a reflection of increased vigilance for Gilberts in patients who have colon cancer. “Tricuspid regurgitation” may be strongly associated to “past use of tobacco” (OR 155.0, p = 1.0×10^−100^) because smoking can cause chronic obstructive pulmonary disease with subsequent development of cardiac disease. “Keloids” and “history of asthma” (OR 17.4, p = 1.1×10^−4^) may have race as a common link, as both conditions are known to occur frequently in African Americans.[13], [14] Finally, “colon cancer” and “osteopenia” (OR 3.9, p = 3.3×10^−27^) may also have a logical explanation. Calcium is thought to prevent adenomas, which can later become colon cancer.[15] Therefore, low calcium may predispose patients to colon cancer, and osteopenia may be a proxy for low calcium levels. Alternatively, ostepenia may also be a side effect of various cancer treatments including chemotherapy and radiation, or from the cancer itself.[16] Knowing the temporal sequence of when the diagnoses were first noted could help point to the cause.

**Table 3:** Associations that are unknown or poorly known but may have an explanation.

Problem	Associated problem	P value	Odds ratio
Shingles	Hypothyroidism	6.2×10^−12^	2.9
Gilberts disease	Family History of colon cancer	2.5×10^−4^	26.5
Keloids	History of asthma	1.1×10^−4^	17.4
Tricuspid regurgitation	Past use of tobacco	1.0×10^−100^	155.0
Colon Cancer	Osteopenia	3.3×10^−27^	3.9

Selected problems for which we do not know of a previously reported association are presented in Table 4. The association between “granuloma annulare” and “osteoarthritis” (OR 4.3, p = 1.1×10^−4^) is interesting since both can be treated with niacin,[17], [18] suggesting that a common underlying pathway might exist. Likewise, the association between “pyloric stenosis” and “ventricular septal defect” (OR 12.1, p = 2.0×10^−3^) is unknown although both are disorders of muscle tissue. Whether or not this suggests a common underlying mechanism is unknown. The associations with “shatskis ring” are also unusual but may be a result of inadvertent findings as a result of radiologic studies.

**Table 4:** Associations that are unknown or poorly known.

Problem	Associated problem	P value	Odds ratio
Granuloma Annulare	Osteoarthritis	1.1×10^−4^	4.3
Granuloma Annulare	Fibromyalgia	1.0×10^−3^	6.9
Pyloric Stenosis	Ventricular Septal Defect	2.0×10^−3^	12.1
Anosmia	Varicella	1.7×10^−18^	46.3
Diverticular disease	Hypothyroidism	1.6×10^−7^	5.4
Attention Deficit Hyperactivity Disorder	Osgood Schlatter disease	1.1×10^−6^	11.5
Irregular Periods	Bipolar disorder	2.0×10^−3^	8.4
Schatzkis ring	Spinal stenosis	4.3×10^−5^	22.3
Schatzkis ring	Diverticulosis	9.8×10^−9^	17.1
Schatzki ring	Nephrolithiasis	3.1×10^−5^	11.0
Irritable bowel syndrome	Plantar fasciitis	8.0×10^−16^	3.5
Breast calcifications	Agoraphobia	8.6×10^−6^	83.7
Thyromegaly	Varicella	8.4×10^−6^	13.6
Fuchs dystrophy	Hypothyroid	1.6×10^−8^	19.8
Cat bite	Depression	1.7×10^−5^	3.0
Ankylosing spondylitis	TURP	1.4×10^−5^	29.4
Spondylolisthesis	Hepatitis A	3.6×10^−5^	23.0
Dacryostenosis	Jaundice	1.6×10^−55^	44.4

This study does have several limitations. Discovering an association does not imply causation and we did not take into account the temporal sequence of the diagnoses. Additionally, simply because an association exists between two diagnoses does not imply medical relevance, nor does it imply that the association is valid. Others who have done similar studies used a threshold for finding relevant associations since some of the weaker ones may simply be due to chance given the large number of comparisons being made.[19] We chose not to ignore less significant associations but rather used our clinical judgment when reviewing them. It may be the case that less significant, but nevertheless real, associations have been overlooked with prior methodologies.

All diagnoses were entered at the discretion of the clinicians in our health system. We do not know if diagnoses were made using strict definitions or classification criteria (e.g., diagnosing a migraine headache when it may really be a tension headache, or diagnosing lupus without use of the 11 criteria). It has been shown that coded diagnoses from billing data can often be extremely inaccurate [20] so it is possible that the diagnoses in our PSL, which are not used for billing purposes, were also inaccurate. Clinicians may also fail to enter all of a patient's problems, which has been reported elsewhere.[21]

The free text nature of the diagnoses in our system also made finding significant associations challenging because some concepts may have been worded differently and not mapped to a single concept. As a result, they would have been considered to be completely different diagnoses by the system. Nevertheless, the large volume of problems did allow us to find significant associations even with the limitation of using free text.

Use of the MCM tool could be useful for hypothesis generation, and the confirmation in recent literature of multiple associations that we found supports this assertion. Further work in the laboratory to elucidate possible mechanisms could confirm the validity of this approach, especially where preliminary reports suggest a common pathway such as the use of niacin to treat both granuloma annulare and osteoarthritis.

We also believe that the significant associations generated could support clinical activities as well. Such a knowledge base could provide a form of clinical decision support to ensure that related diagnoses are not missed, or even to support the entry into the PSL of related problems that a clinician may not have thought to enter into the EHR. Furthermore, the knowledge base could be continually and automatically updated as more data are entered into the PSL by clinicians.

It might be possible, for example, that if someone were to enter “low back pain” as a diagnosis (see Table 1) that such a system could prompt the clinician to also ask about problems with “insomnia” since the association was strong. Insomnia may be a result of both the suffering one endures from chronic back pain as well as from possible treatments for back pain [22], [23] but it would be important for a clinician to consider the possibility of a sleep disorder in someone with back pain.

Future work with this tool could involve implementing in a clinical care setting a system loaded with the associations to provide real-time suggestions to clinicians to determine the utility of the suggestions. We also believe that comparing our results with those of other institutions would help to support or refute some of the more unusual findings uncovered in our analysis. Furthermore, combining clinical diseases with laboratory findings, such as what was done with the Human Disease Network,[24] could further help uncover and elucidate novel associations.
